# Testing the Transportability of the Psychosis Metabolic Risk Calculator in Canada (Quebec): International External Validation Study

**DOI:** 10.1093/schbul/sbaf174

**Published:** 2025-10-27

**Authors:** Sébastien Brodeur, Olivier Corbeil, Laurent Béchard, Maxime Huot-Lavoie, Charles Desmeules, Dominic Oliver, Andrea De Micheli, Emanuele F Osimo, Rachel Upthegrove, Golam M Khandaker, Graham K Murray, Fanny Lajeunesse-Trempe, Gloria Cheung, Josiane Courteau, Chantale Thériault, Marc-André Roy, Marie-France Demers, Benjamin I Perry

**Affiliations:** Department of Psychiatry and Neuroscience, Faculty of Medicine, Laval University, Quebec, G1V 0A6, Canada; CERVO Brain Research Centre, Quebec, G1E 1T2, Canada; Early Psychosis: Interventions and Clinical-Detection (EPIC) Lab, Department of Psychosis Studies, Institute of Psychiatry, Psychology & Neuroscience, King’s College London, London, SE5 8AF, United Kingdom; University Institute in Mental Health of Quebec, Quebec, G1J 2G3, Canada; CERVO Brain Research Centre, Quebec, G1E 1T2, Canada; University Institute in Mental Health of Quebec, Quebec, G1J 2G3, Canada; Faculty of Pharmacy, Laval University, Quebec, G1V 0A6, Canada; CERVO Brain Research Centre, Quebec, G1E 1T2, Canada; University Institute in Mental Health of Quebec, Quebec, G1J 2G3, Canada; Faculty of Pharmacy, Laval University, Quebec, G1V 0A6, Canada; CERVO Brain Research Centre, Quebec, G1E 1T2, Canada; University Institute in Mental Health of Quebec, Quebec, G1J 2G3, Canada; CERVO Brain Research Centre, Quebec, G1E 1T2, Canada; University Institute in Mental Health of Quebec, Quebec, G1J 2G3, Canada; Early Psychosis: Interventions and Clinical-Detection (EPIC) Lab, Department of Psychosis Studies, Institute of Psychiatry, Psychology & Neuroscience, King’s College London, London, SE5 8AF, United Kingdom; Department of Psychiatry, University of Oxford, Oxford, OX3 7JX, United Kingdom; NIHR Oxford Health Biomedical Research Centre, Oxford, OX3 7JX, United Kingdom; OPEN Early Detection Service, Oxford Health NHS Foundation Trust, Oxford, OX3 7JH, United Kingdom; Early Psychosis: Interventions and Clinical-Detection (EPIC) Lab, Department of Psychosis Studies, Institute of Psychiatry, Psychology & Neuroscience, King’s College London, London, SE5 8AF, United Kingdom; OASIS Service, South London and Maudsley NHS Foundation Trust, SE11 5DL, United Kingdom; Department of Psychiatry, University of Cambridge, Cambridge, CB20SZ, United Kingdom; Cambridgeshire and Peterborough NHS Foundation Trust, CB21 5EF, United Kingdom; Department of Psychiatry, University of Oxford, Oxford, OX3 7JX, United Kingdom; Institute for Mental Health, University of Birmingham, Birmingham, B15 2TT, United Kingdom; Bristol Medical School, University of Bristol, Bristol, BS8 1UD, United Kingdom; Department of Psychiatry, University of Cambridge, Cambridge, CB20SZ, United Kingdom; Cambridgeshire and Peterborough NHS Foundation Trust, CB21 5EF, United Kingdom; Quebec Heart and Lung University Institute, Laval University, Quebec, G1V 4G5, Canada; Hull York Medical School, York, YO10 5DD, United Kingdom; York and Scarborough Teaching Hospitals NHS Foundation Trust, York, YO31 8HE, United Kingdom; PRIMUS Research Group, Research Centre of the Sherbrooke University Hospital, Sherbrooke, J1H 5H3, Canada; CERVO Brain Research Centre, Quebec, G1E 1T2, Canada; University Institute in Mental Health of Quebec, Quebec, G1J 2G3, Canada; CERVO Brain Research Centre, Quebec, G1E 1T2, Canada; University Institute in Mental Health of Quebec, Quebec, G1J 2G3, Canada; Faculty of Pharmacy, Laval University, Quebec, G1V 0A6, Canada; CERVO Brain Research Centre, Quebec, G1E 1T2, Canada; University Institute in Mental Health of Quebec, Quebec, G1J 2G3, Canada; Faculty of Pharmacy, Laval University, Quebec, G1V 0A6, Canada; Institute for Mental Health, University of Birmingham, Birmingham, B15 2TT, United Kingdom

**Keywords:** First-episode psychosis (FEP), Metabolic syndrome (MetS), risk prediction model, external validation, North-America

## Abstract

**Background and Hypothesis:**

Cardiometabolic morbidity largely explains premature mortality in people with psychotic disorders and is detectable from psychosis onset. Currently, no accurate cardiometabolic risk prediction tool exists for young people with first-episode psychosis (FEP). The Psychosis Metabolic Risk Calculator (PsyMetRiC) aims to bridge this gap, but its accuracy and potential clinical usefulness in North American populations remain unverified.

**Study Design:**

The external validity of PsyMetRiC, developed in the United Kingdom to predict the risk of incident metabolic syndrome (MetS) up to 6 years after a FEP, was assessed using the data from the Quebec Psychosis Early Intervention Clinic. PsyMetRiC comprises 2 penalized logistic regression models: a full-model including age, sex, ethnicity, body mass index (BMI), smoking status, prescription of metabolically-active antipsychotic medication, high-density lipoprotein (HDL), and triglyceride concentrations; and a partial-model excluding biochemical predictors. Patients aged 16-35 years, diagnosed with FEP between 2004 and 2023 without pre-existing MetS, and with>12 months follow-up were included. Predictive performance of PsyMetRiC was assessed by discrimination (C-statistic), calibration (calibration plots), and clinical usefulness (decision curve analysis). The race and ethnicity predictor was refined to better represent the North American population.

**Study Results:**

Among 559 included patients (mean age 24.1 years ±4.1; 22.5% female), 18.2% developed MetS during a mean follow-up of 1.7 ± 1.3 years. Compared with the UK development cohort, the Canadian sample exhibited a higher BMI, lower HDL cholesterol, lower triglycerides, lower blood glucose, and lower systolic blood pressure. Discrimination performance was acceptable (full model C = 0.74, 95% CI, 0.70-0.77; intercept = 0.225; slope = 1.278; partial model C = 0.70, 95% CI, 0.67-0.74; intercept = −0.555; slope = 0.993). After updating the model with a race and ethnicity predictor calibrated to locally representative categories, performance improved slightly (full model C = 0.74, 95% CI, 0.71-0.77; intercept = 0.000; slope = 1.001; partial model C = 0.71, 95% CI, 0.68-0.74; intercept = 0.001; slope = 1.005).

**Conclusions:**

This study provides the first external validation of PsyMetRiC in a North American sample. Further research is essential before routine clinical implementation, but PsyMetRiC offers promise as a tool for early detection of cardiometabolic risk in early psychosis, guiding personalized treatments to diminish long-term physical health impacts.

## Introduction

People with psychotic disorders have a life expectancy 15-20 years shorter than the general population, predominantly explained by physical comorbidities such as cardiovascular disease (CVD), diabetes, and obesity.^1^^,^^2^ Attributions include several cardiovascular risk factors which are commonly present in people with psychotic disorders, such as a sedentary lifestyle, unhealthy diet, smoking, and disrupted sleep.^3^ In addition, some routinely prescribed antipsychotics cause weight gain and metabolic adverse effects, regardless of their therapeutic benefits.^4^ Although these metabolic effects are common, there is considerable variability in how they affect each individual.^5^

Metabolic syndrome (MetS) is an early marker of cardiometabolic morbidity and mortality and is defined by a cluster of cardiometabolic features, including impaired glucose and insulin homeostasis, abdominal obesity, hypertension, and dyslipidemia.^6^ MetS significantly increases the risk of developing diabetes, with a 4-fold increase in relative risk; and doubles the risk of CVD and premature mortality in the general population,^7^ with similar results observed in schizophrenia.^8^ In addition, an increased prevalence of MetS has been observed in patients with first-episode psychotic disorders (FEP) prior to antipsychotic exposure.^9^ Indeed, the high-pooled global prevalence of MetS in people with schizophrenia highlights the widespread nature of this cardiometabolic risk in this population.^10^ However, there are some differences between countries and continents.^10^ When considering severe mental disorders, including schizophrenia and bipolar disorder, the reported MetS prevalence was 27.4% in Canada, 36.4% in the United States, 32.4% in North America, and 32% in Europe,^10^ though it is acknowledged that patients with schizophrenia are under-screened and under-treated for their cardiovascular conditions.^3^

The possibility of early detection of MetS represents an important opportunity to prevent downstream adverse cardiometabolic outcomes, such as CVD, in people with psychotic disorders. Meeting these challenges requires innovative strategies for both early risk detection and proactive management. On the former, the Psychosis Metabolic Risk Calculator (PsyMetRiC) is one such innovation.^11^ Developed in the United Kingdom and first published in 2021, PsyMetRiC is the first cardiometabolic risk prediction algorithm specifically designed for young people with psychotic disorders. PsyMetRiC predicts future MetS up-to 6-years in advance from FEP using only routinely collected information at baseline. Following external validation in the United Kingdom, then in Spain,^12^ Switzerland,^12^ Hong Kong,^13^ Finland,^14^ and Australia,^15^ PsyMetRiC has shown good evidence of potential transportability and likely clinical usefulness across populations in predicting the risk of MetS up to 6 years after baseline. These studies reported consistent and generally acceptable to good discriminative performance (C-statistics for the full model generally ranging from 0.72 to 0.76), with some variation in calibration that was improved through local recalibration. For instance, the model demonstrated robust performance in the Swiss (C = 0.73) and Spanish (C = 0.72) cohorts, as well as in the Hong Kong cohort (C = 0.76). The degree of clinical usefulness varied depending on local baseline risk and population characteristics. In Finland, the model also generalized well (C = 0.72), despite the absence of ethnicity data due to population homogeneity. In the Australian validation study, ethnicity data were unavailable; instead, a proxy variable indicating whether individuals were born in Australia or overseas was used to approximate population diversity. However, the transportability of any predictive algorithm to different geographical populations cannot be assumed, since population differences in, for example, culture, health behaviors, and health systems may impact generalizability. In this respect, the external validity of PsyMetRiC has not been assessed in North America; hence generalizability cannot be assumed for North American samples. The high prevalence of MetS in our population, combined with a high prevalence of this syndrome in the North American psychiatric population, as well as the lack of validated evidence for the management of our patients in Quebec (Canada), highlight the necessity of such an evaluation.

Therefore, we aimed to evaluate the predictive performance of PsyMetRiC in a sample of young people aged 16-35 years with FEP attending an early intervention service located in Quebec, Canada. Furthermore, we explored recalibration and revision approaches for the model, specifically updating the race and ethnicity predictor, to improve its alignment with the demographic characteristics of the North American population.

## Methods

### Study Design, Setting, and Population

This retrospective longitudinal study used data collected from an early intervention service for FEP in the metropolitan area of Quebec City (≈760 000 residents), Canada. We followed recently updated gold-standard TRIPOD-AI guidelines^16^ (Supplementary Table 6). A protocol was internally prepared for submission to the ethical review panel (available on request). Study preregistration was not completed. The clinic provides care for patients with FEP, including both affective (ie, depression and bipolar disorder with psychotic features) and non-affective (ie, schizophrenia spectrum) psychotic disorders, typically aged 18-35 years. However, depending on clinical circumstances, patients aged 16-37 may occasionally be seen. For this study, and in line with the original PsyMetRiC validation criteria, we included only patients aged 16-35 years. This age range was applied consistently throughout the analysis to ensure comparability with previous PsyMetRiC studies. The inclusion criteria for the service include use of antipsychotic medication for no more than 6 months, and no concurrent diagnosis of DSM-5 moderate or severe intellectual disability. Individuals admitted to the clinic are followed for up to 3 years using a case management approach.

The study cohort included all patients admitted to the clinic between 2004 and 2023 and who met the following criteria, per the original PsyMetRiC study^11^: at least 12 months of follow-up; a diagnosis of psychosis-spectrum disorder (ICD-10 codes F06.0-2, F20-F31, F32.3, F33.3, F53.1); and without MetS at baseline. Patients with missing data on all predictors or outcome constituent variables were excluded.

### Outcome

As per the original PsyMetRiC study,^11^ the harmonized definition of MetS as a binary outcome was used^17^: race and ethnicity-specific waist circumference ≥94 cm in males or ≥80 cm in females for Caucasians; ≥90 cm in males or ≥80 cm in females for other race and ethnicity groups, or body mass index (BMI) > 29.9; alongside 2 of: triglycerides ≥1.70 mmol/L; high-density lipoprotein (HDL) <1.03 mmol/L in males or < 1.29 mmol/L in females; systolic blood pressure > 130 mmHg; fasting plasma glucose >5.60 mmol/L. Where multiple follow-ups were available for each participant, we used the latest follow-up available between 1 and 6 years after baseline with the least amount of missing data.

### PsyMetRiC and Predictor Variables

PsyMetRiC consists of 2 forced-entry multivariable penalized logistic regression models: the full model and the partial model. Predictor variables were selected based on clinical relevance, prior research, and their potential utility in routine care, consistent with the original PsyMetRiC development study. Acceptability was assessed by engaging with all key stakeholders including, young people with lived experience of psychosis, clinicians, carers, and family members (see https://mcpin.org/project/the-psychosis-metabolic-risk-calculator-project/). The full model includes age (continuous, in years), sex assigned at birth (male or female), BMI (continuous, in kg/m^2^), smoking status (binary; defined as current smoker if smoking at least one cigarette per day), prescription of a metabolically active antipsychotic medication (binary; based on relative cardiometabolic risk), HDL cholesterol (continuous, in mmol/L), and triglyceride concentration (continuous, in mmol/L). The partial model includes all of the above variables except HDL cholesterol and triglycerides, to accommodate settings where biochemical data may not be available. To enhance the model’s relevance for a North American population, the race and ethnicity predictor was refined from the original UK-based categories. In this study, race and ethnicity were recoded as follows: White, Black or African American, East Asian or Aboriginal, Hispanic or Latino, South Asian, or Other. This modification was made in accordance with updated guidance on demographic reporting in medical research and reflects the diversity of the Canadian population.^18^ Race and ethnicity variables were recoded based on the original variable, which was recorded in clinical health records and assigned by clinicians, rather than being self-reported by patients. All continuous variables were measured using standard clinical units. Further details on the original PsyMetRiC coefficients are provided in Supplementary Table 5. See the original PsyMetRiC study for further details.^11^

### Statistical Analyses

#### Sample Preparation and Estimation of Analytic Precision

Using published formulas, we estimated the precision of our analyses based on the fixed available sample size.^19^ Briefly, with inputs of the estimated outcome prevalence (20%) and anticipated C-statistic (0.74) based on previous PsyMetRiC studies, the available sample size would enable standard errors for the C-statistic and calibration slope of 0.03 and 0.13, respectively. In cases of missing data, multiple imputation using chained equations was considered for variables with <50% missingness and the presence of auxiliary variables to reduce the fraction of missing information (see Supplementary methods). For numerical-based analyses, estimates were pooled using Rubin’s rules. For plot-based analyses, plots were generated in each imputed dataset and checked for similarity, with one randomly selected plot per analysis presented in the main manuscript and all remaining plots presented in the Supplementary data. The original UK PsyMetRiC development sample was compared with the Quebec, Canada analytic sample on key sociodemographic, lifestyle, and biochemical characteristics using t-tests for means and chi-square equality of proportions tests for proportions.

#### Primary External Validation Analysis

PsyMetRiC was applied to the analytic sample and the distribution of predicted outcome probabilities was examined using histograms. Predictive performance was assessed primarily using measures of discrimination (concordance [C-] statistic), calibration (calibration plots), and clinical usefulness (decision curve analysis, see Supplementary methods) as recommended. In addition, we recorded the calibration intercept (ideally close to 0), the calibration slope (ideally close to 1), and the Brier score (ideally close to 0).

#### Recalibration and Updating with Refined Race and Ethnicity Predictor to Generate Canada-specific PsyMetRiC Version (PsyMetRiC-Can)

PsyMetRiC was updated by re-estimating the race and ethnicity predictor to more closely reflect the racial and ethnic composition of the study population, in line with guidance^18^ and based on available data. The new predictor was coded as White, Black or African American, East Asian or Aboriginal, Hispanic or Latino, South Asian, or Other. To do this, we fit a logistic regression model in the sample with the linear predictor from the primary external validation analysis alongside the new race and ethnicity predictors. After extracting averaged intercept and slope coefficients for the recalibrated model, heuristic shrinkage^20^ was applied to account for over-optimism of the newly estimated predictors. The heuristic shrinkage factor is defined as (model χ^2^ − df/model χ^2^), where model χ^2^ refers to the difference in −2 log-likelihood between a model with and without predictors, and df refers to the degrees of freedom used by the predictors.^20^ The individual linear predictors and predicted probabilities for each participant were then recalculated using the updated intercept, slope, and new variable coefficients. The predictive performance was reassessed. By completing this step, we developed a Canadian-specific version of PsyMetRiC (PsyMetRiC-Can).

### Ethics

Patients included in this study provided prior informed consent for their denominative sociodemographic and clinical data to be collected, stored in a secure research database, and used for research purposes (ethical approval #222-2009). This study received ethical approval (#2024-2963) and met local requirements pertaining to data management and confidentiality from the Research Ethics Board for Neurosciences and Mental Health of the Centre intégré universitaire de santé et de services sociaux de la Capitale-Nationale.

### Role of the Funding Source

The funders of this study had no role in study design, data collection, data analysis, data interpretation, or writing of the report.

## Results

### Characteristics of the Sample

A total of 661 potentially eligible patients were considered for inclusion in the study, of whom, 559 (84.5%) met all inclusion criteria and formed the analytic sample. Five participants were excluded based on age, and 97 (14.8%) were excluded due to meeting criteria for MetS at baseline; a proportion significantly higher than that observed in the original UK PsyMetRiC development sample (4.6%, *P* < .001). No participants were excluded due to missing data. The analytic sample also differed in several sociodemographic, lifestyle, and biochemical characteristics compared with the original UK sample (Table 1). The PsyMetRiC-Can cohort was predominantly male (77.5%) with a mean age of 24.1 years (SD 4.1) and a mean follow-up period of 1.74 years (SD 1.29). The racial and ethnic composition of the Canadian cohort, when characterized using the criteria of the original PsyMetRiC study, was different than the UK sample, with a higher proportion of White individuals and lower proportions of Black/African-Caribbean and Asian/Other individuals than observed in the UK cohort. Compared with the UK cohort, the Canadian cohort had higher BMI, lower HDL, lower triglycerides, lower blood glucose, and lower systolic blood pressure at baseline. There were no statistically significant differences in the use of more metabolic antipsychotics and smoking status at baseline, in the follow-up time, or in the proportion of patients with incidental MetS during follow-up.

**Table 1:** Socio-demographic Characteristics of the Original PsyMetRiC Development Sample and the Analytic Sample.

**Characteristic**	**Canada validation sample** **(*n* = 559)**		**Original PsyMetRiC development sample (UK)** **(*n* = 651)**		***P*-value**
Age, years, mean (SD)	24.1	(4.09)	24.5	(4.91)	.128
Race and ethnicity based on PsyMetRiC coding, *N* (%)					
White European	447	(80.0)	360	(55.3)	
Black/African-Caribbean	54	(9.7)	109	(16.7)	
Asian/Other	58	(10.4)	181	(27.8)	
Male sex, *N* (%)	433	(77.5)	440	(67.6)	**<.001**
HDL at baseline, mmol/L, mean (SD)	1.38	(0.41)	1.88	(0.57)	**<.001**
Triglycerides at baseline, mmol/L, mean (SD)	1.25	(0.77)	1.39	(1.06)	**.010**
Waist Circumference at baseline, cm (SD)	89.1	(11.6)	–	–	–
BMI at baseline, kg/m^2^, mean (SD)	25.1	(4.10)	23.63	(5.43)	**<.001**
FPG at baseline, mmol/L, mean (SD)	4.93	(0.54)	5.19	(1.28)	**<.001**
Systolic BP at baseline, mmHg, mean (SD)	118	(11.5)	120.65	(11.68)	**<.001**
Prescribed a More-Metabolically-Active Antipsychotic^a^, *N* (%)	377	(67.4)	455	(69.9)	.646
Smoking at baseline, *N* (%)	265	(47.4)	315	(48.4)	.977
Follow-up time, years, mean (SD)	1.74	(1.29)	1.86	(1.32)	0.111
Metabolic Syndrome during Follow-up, *N* (%) (post imputation)	96	(17.2)	109	(16.7)	0.464

Abbreviations: HDL = high-density lipoprotein; BMI = body mass index; FPG = fasting plasma glucose; BP = blood pressure.

a Definitions of metabolically active antipsychotics are given in Supplementary Table 3.

Analysis of means was conducted using t-tests. Analysis of proportions were conducted using chi-square equality of proportions tests.

### Primary External Validation Analysis

The distribution of PsyMetRiC predicted probabilities in the analytic sample was similar to the original UK PsyMetRiC study (Supplementary Figure 1). Predictive performance statistics are detailed in Table 2. Discrimination performance was adequate (C = 0.74, 95% 95%CI, 0.71-0.77 for the full model, and C = 0.71, 95%CI, 0.68-0.74 for the partial model). Calibration plots for the full model were similar across imputed datasets (Figure 1, Supplementary Figure 2) and show a systematic degree of risk overprediction. For the partial-model, calibration plots were similar across imputed datasets and show overprediction of risk at higher predicted probabilities (Figure 1, Supplementary Figure 2).

**Table 2:** Predictive Performance Statistics of the PsyMetRiC Full and Partial Models Before and After Logistic Calibration in the PsyMetRic-Can Sample

**Measure of predictive performance**	**Primary analysis, estimate (95% CI)**		**After updating and recalibration, estimate (95% CI)**	
	**Full-model**	**Partial-model**	**Full-model**	**Partial-model**
**C-Statistic**	0.74 (0.70, 0.77)	0.70 (0.67, 0.74)	0.74 (0.71, 0.77)	0.71 (0.68, 0.74)
**Calibration Intercept**	0.225 (0.13, 0.32)	−0.555 (−0.66, −0.46)	0 (−0.00, 0.00)	0.001 (0.001, 0.001)
**Calibration Slope**	1.278 (1.10, 1.45)	0.993 (0.84, 1.15)	1.001 (0.997, 1.005)	1.005 (0.997, 1.013)
**Brier Score**	0.13 (0.12, 0.14)	0.142 (0.13, 0.15)	0.127 (0.12, 0.14)	0.132 (0.12, 0.14)

The C-statistic is a measure of discrimination and estimates the probability that a randomly selected “case” has a higher predicted probability than a randomly selected non-case. Values of 1.0 indicate perfect discrimination; values >0.70 are generally considered acceptable. The calibration intercept (ideally close to 0) and calibration slope (ideally close to 1) are estimates of the model calibration (ie, the agreement between the observed proportion and the predicted risk). The Brier score (ideally close to 0, with scores >0.25 indicating poor performance) is an overall measure of algorithm performance. For comparison, the results of the original external validation of PsyMetRiC in the UK were: full model C = 0.75 (95%CI, 0.69-0.80); Brier score = 0.07 (95%CI, 0.04-0.10); intercept = -0.05 (95%CI, −0.08 to −0.02); partial model: C = 0.74 (95%CI, 0.67-0.79); Brier score = 0.08 (95%CI, 0.05-0.11); intercept = −0.07 (95%CI, −0.11 to −0.03). See original PsyMetRiC manuscript for further details.^11^

**Figure 1:**
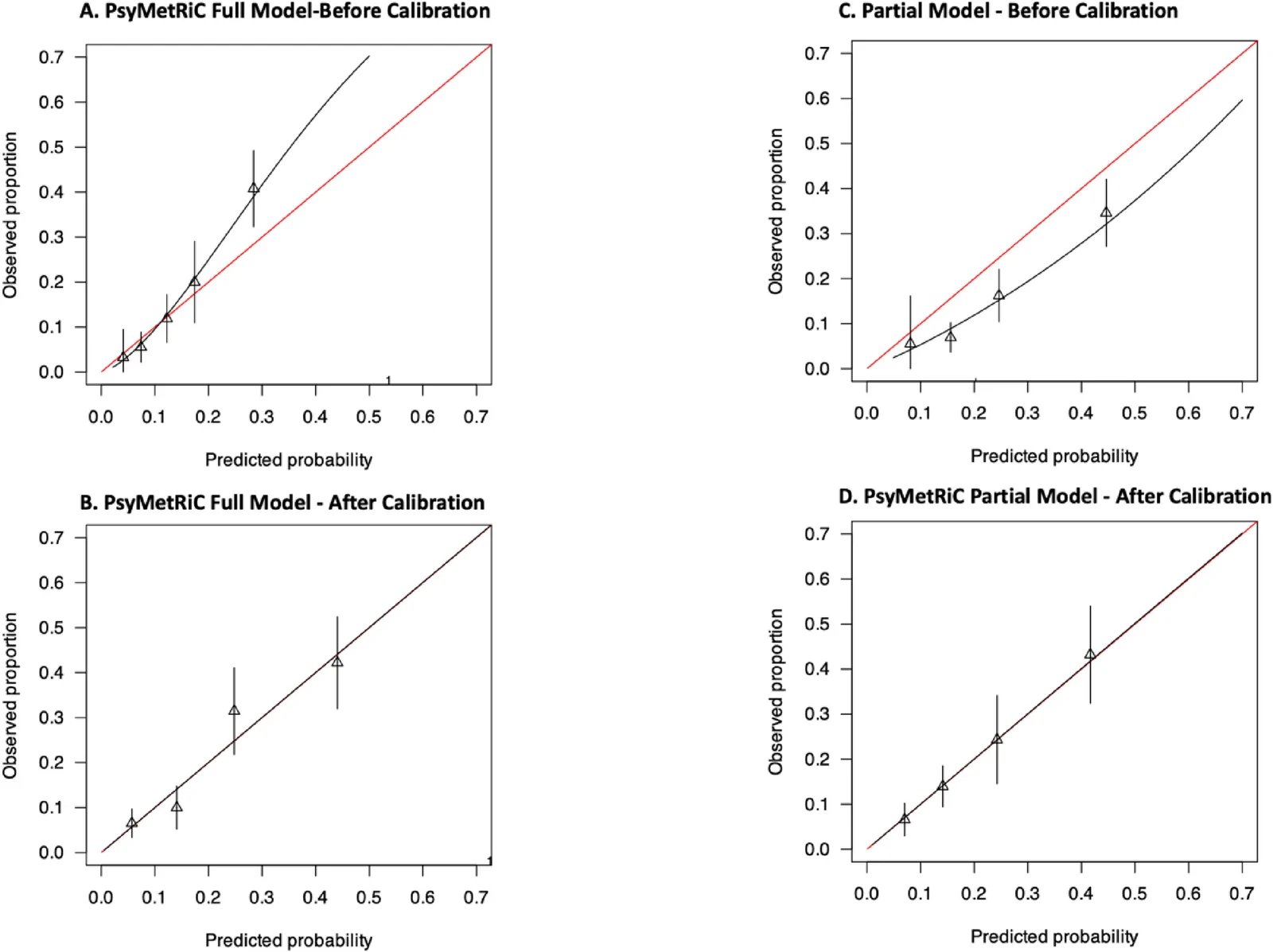
Calibration plots of PsyMetRic in PsyMetRiC-Can sample. (A) Primary analysis—full model; (B) after logistic calibration—full model; (C) primary analysis − partial model; (D) after logistic calibration—partial model. The calibration plots show the agreement between the observed risk (*y*-axis) and the predicted risk (*x*-axis). Perfect agreement would be shown by the red line. The calibration of the algorithm is shown by the black line. Triangles indicate grouped observations for participants at deciles of predicted risk, with 95% CIs indicated by the vertical black lines. Logistic calibration accounts for differences in baseline risk that may exist between populations by re-estimating the intercept term, and also re-estimates the slope term, thus assuming similar relative effects of the predictors but allowing for a larger or smaller absolute effect of the predictors.

### Algorithm Updating, Recalibration, and Generation of Site-specific PsyMetRiC version (PsyMetRiC-Can)

We re-estimated the race and ethnicity predictor to better represent the local population. Based on the available data, race and ethnicity were recoded as follows: White (*n* = 447), Black or African American (*n* = 54), Hispanic or Latino (*n* = 14), South Asian (*n* = 15), East Asian/Aboriginal (*n* = 18), and Other (*n* = 11). After updating PsyMetRiC with the new race and ethnicity predictor and recalibrating the intercept and slope terms (Table 2, Supplementary Table 4), the shape of the distributions of predicted probabilities was similar to the primary analysis (Supplementary Figure 1). The recalibrated performance statistics are reported in Table 2. Discrimination improved slightly in both PsyMetRiC versions (Full-model: C = 0.74, 95%CI, 0.71-0.77; Partial-model: C = 0.71, 95%CI, 0.68-0.74). The calibration plots for both PsyMetRiC versions were similar across imputed datasets (Figure 1, Supplementary Figure 2) and showed improved calibration performance.

### Clinical Usefulness

The decision curve analysis (Figure 2, Supplementary Figure 3) showed that in both partial and full models, PsyMetRiC-Can provided universally greater net benefit than competing strategies. The net benefit was greater with the full model compared to the partial model. For example, in the analytic sample, if an intervention was considered for participants scoring higher than 0.15, the recalibrated full and partial models provided net benefits of 0.077 (95%CI, 0.05-0.11) and 0.062 (95%CI, 0.03-0.09) respectively, meaning that an additional 46% of MetS cases could be prevented with the full model, and 36% with the partial model, with no increase in false positives.

**Figure 2:**
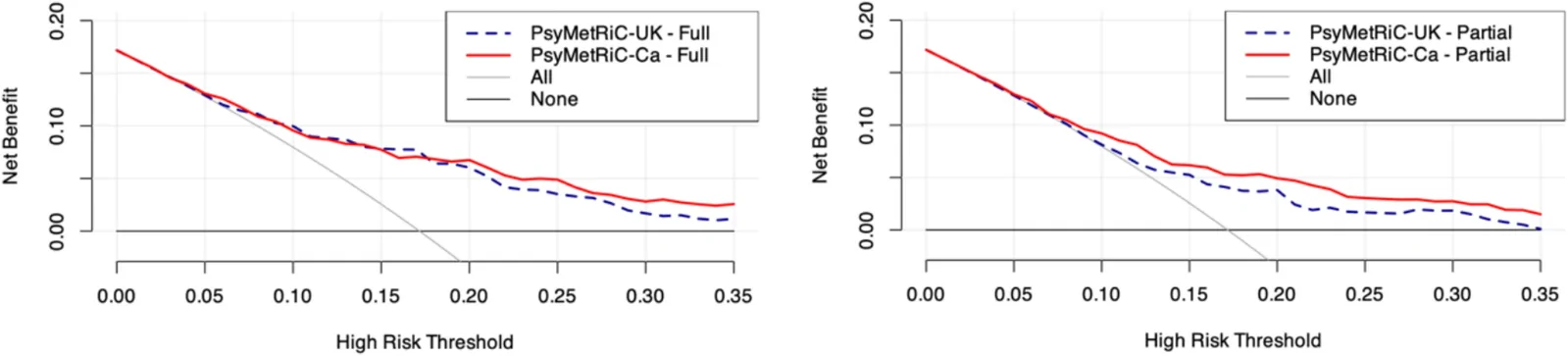
Clinical usefulness of PsyMetRiC in the PsyMeTriC-Can sample before and after logistic calibration. The plot shows the net benefit (*y*-axis) of the PsyMetRiC-can full and partial models (blue dotted line = original PsyMetRiC algorithm applied to the sample; red solid line = recalibrated site-specific version) across a range of risk thresholds (*x*-axis) compared with intervention in all (gray solid line) or intervention in none (black solid line). In decision curve analysis, it is common practice to only consider the range of risk thresholds that can reasonably be taken into account in clinical practice. Our upper limit of 0.30 represents about a one in 3 chance of developing MetS if nothing changes, and it is unlikely that higher risk thresholds would be tolerated. Net harm (ie, more false positives than true positives exposed to an intervention at a chosen risk threshold) is indicated when the decision curve line is plotted at *y* < 0.

## Discussion

This study represents the first evaluation of the PsyMetRiC cardiometabolic risk prediction algorithm in North America, a critical step toward its eventual integration into routine clinical practice. The validation analyses, conducted in a large cohort of young people experiencing FEP, showed that the discriminative performance of PsyMetRiC, as assessed by the C-statistic, closely mirrored that of the original UK study and subsequent external validation tests, for both the full and partial models.^12^ Consistent with previous research,^12^^,^^13^^,^^21^ the full model showed slightly better performance than the partial model in discriminating cases of MetS. This highlights the importance of biochemical assessment in appraising cardiometabolic risk in young people with psychosis. Nevertheless, both models showed adequate performance. Although the full model should be favored when possible, the partial-model is also likely to be clinically useful. This is underlined by the greater net benefit of its use compared with the 2 extremes of either intervening in all patients or in none, and across a wide range of potential risk thresholds.

The calibration of the models represents the agreement between the predicted and the observed risk. Thus, it is crucial to the clinical usefulness of PsyMetRiC in the future, since over- or under-prediction of risk may impact the appropriate distribution of interventions if risk thresholds for specific interventions are introduced. The minor miscalibration observed in both the original PsyMetRiC versions at higher predicted probabilities may be due in part by differences between our sample and that of the original study, but reflect the need for population-specific alternations to maximize the accuracy of PsyMetRiC locally. In the present study, calibration plots for the full model were consistent across all imputed datasets, demonstrating a systematic tendency to overpredict risk. Similarly, the partial model exhibited comparable calibration patterns across imputation, with notable overprediction at higher predicted probabilities. These calibration issues are not unique to our analysis. Similar patterns have been reported in other external validations of PsyMetRiC, as well as in assessments of widely adopted cardiovascular risk prediction tools such as the Framingham Risk Score.^22^^,^^23^ These general population-based algorithms have consistently demonstrated variable calibration performance across different populations, often overestimating or underestimating risk depending on demographic, clinical and regional factors. For example, studies have shown that the Framingham model tends to overpredict risk in low-risk populations and women, while underpredicting in certain high-risk groups.^22^^,^^23^ Such discrepancies highlight the limitations of applying a single model universally and help to explain the variation in calibration observed in our study. This underlines the importance of local recalibration and population-specific adaptation to ensure accurate and equitable risk prediction.

For example, PsyMetRiC was initially developed with a race and ethnicity predictor tailored to the UK population. To improve its relevance to North American populations, the race and ethnicity predictor was re-estimated to reflect the demographics of the study population. After this update, the distribution of the predicted probabilities remained consistent with the primary analysis, indicating that the prediction model’s core structure was retained. Importantly, this methodological step resulted in slight improvements in discrimination for both the full model (C = 0.74, 95%CI, 0.71-0.77) and the partial model (C = 0.71, 95%CI, 0.68-0.74). The calibration plots showed improved calibration performance across imputed datasets, suggesting improved accuracy of predicted probabilities. However, a significant limitation of our study is the small sample size of non-white individuals. This highlights the need for further updating and validation in larger and more diverse samples to ensure equitable model performance across different racial and ethnic groups.

The present sample differed significantly in a number of key characteristics and was more representative of the North American population as a whole. For instance, the baseline prevalence of MetS in this study was more than twice that of the previously studied samples, including the original PsyMetRiC sample; the baseline BMI of the included patients was also higher in this North American sample, with lower HDL levels. These differences could reflect varying diets, lifestyles, and genetic backgrounds. In addition, the higher prevalence of MetS at baseline could also partly result from a higher use of antipsychotics in Canada, as reported in a previous study that found a significant geographic variation; in 2019, antipsychotic exposure (defined daily dose per 1000 inhabitants per day) was 14.26 in Canada and 10.45 in the United Kingdom.^24^ Nevertheless, the results of this study suggest that PsyMetRiC may be generalizable to at least some regions of North America with relatively similar socioeconomic and environmental settings to those of the present study.

Beyond the performance of PsyMetRiC observed in this study, the results also call for a discussion on how PsyMetRiC may be used in clinical practice. Current guidelines highlight the need for monitoring cardiometabolic health in individuals with psychotic disorders, with an emphasis on early intervention to prevent CVD and related mortality. Despite recognizing this need, details regarding the most effective timing and nature of such interventions remain unclear. Questions remain about when clinicians should be concerned about cardiometabolic health, considering switching to a more neutral antipsychotic, or avoiding initiation of a more cardiometabolically-active antipsychotic altogether.

For instance, in North America, guidelines advocate careful monitoring of the physical and metabolic health of patients diagnosed with psychosis-spectrum disorders, particularly those undergoing antipsychotic treatment.^25-28^ In Canada, guidelines suggest that these patients should maintain a healthy diet and regular physical activity as advised by mental health care providers and undergo regular assessments of weight and cardiovascular and metabolic health.^25^ They also emphasize the importance of lifestyle interventions, including exercise, dietary modification and behavioral strategies, especially for patients who experience weight gain from antipsychotic medications, with metformin as a highlighted recommendation. However, the transition from a recommendation to practice in monitoring and implementing lifestyle interventions after antipsychotic prescription needs improvement. A proactive approach is essential to reduce the increased risk of metabolic and cardiovascular morbidity and mortality in people with schizophrenia beyond their treatment regimen. While the benefits of physical activity and a healthy diet are recognized, effective implementation strategies are scarce. The US guidelines provide a more general perspective on managing MetS in patients with psychosis and schizophrenia, recognizing the increased physical disturbances but lacking specific management strategies.^26^ They call for regular monitoring of metabolic parameters, but fall short in detailing the interventions and follow-up required for effective management. Even when recommendations are clear, patients do not always receive the necessary attention and screening. Studies have shown that many patients with metabolic disorders associated with antipsychotic use do not receive appropriate treatment, highlighting a gap in screening and follow-up.^29^ This situation is partly due to a lack of clarity about responsibility for monitoring metabolic abnormalities between physical and mental health care providers.^30^ As a result, guidelines for the management of people at risk of MetS are not well defined, and PsyMetRiC could be a valuable research tool in helping to address this challenge, by identifying young people at high risk of MetS at baseline so that personalized interventions could be recommended based on the abnormalities detected.

This study has a number of strengths: to begin with, it was the first PsyMetRiC validation study conducted in North America. The results are comparable to those of the original studies, suggesting the potential transportability of PsyMetRiC to North American populations. An additional strength of the study is the minimal amount of missing data. Specifically, the proportion of missing data was less than 5% for all sociodemographic variables, including sex, age, race/ethnicity, smoking status, and antipsychotic prescription. This is due to the quality of the database and the ongoing monitoring of missing data by research and clinical teams. However, there are a number of limitations of the study. First, the analysis was conducted using data from a single Canadian Centre, thus further validation studies in Canada and the rest of North America are required. Nevertheless, several studies have shown that there are similarities in the characteristics of the Canadian population in FEP clinics,^31^^,^^32^ suggesting potential broader generalizability across Canada. Second, our recalibration analysis may be limited by the relatively small number of non-White participants, and the relatively low proportion of females in the analytic sample. Future PsyMetRiC validation and updating studies in North America require more diverse samples. Third, while our sample size is the largest of any PsyMetRiC validation study conducted to date, analytic precision could be improved further with even larger sample sizes in future.

## Conclusion

This study reports an external validation of the PsyMetRiC algorithm in a North American sample and highlights its potential utility in the early detection of cardiometabolic risk in young people with psychosis. PsyMetRiC may facilitate early intervention by identifying patients at risk of poor physical outcomes, enabling personalized care and promoting integration of care between mental health and primary care services. Although further validation is needed prior to routine clinical use, PsyMetRiC has the potential to guide stratified pharmacological and non-pharmacological interventions to reduce long-term morbidity and mortality in psychotic disorders. Importantly, further research in more demographically diverse North American populations will be valuable to confirm and extend the generalizability of these findings.

## Supplementary Material

PsyMetRiC_Can-SupMat-25052025_sbaf174

## Data Availability

The participants of this study did not give written consent for their data to be shared publicly, so due to the sensitive nature of the research supporting data is not available. Statistical and analytic code is available upon request.
