# Supplementary material for: Testing the Transportability of the Psychosis Metabolic Risk Calculator in Canada (Quebec): International External Validation Study
Source: Schizophr Bull. 2025 Oct 27;52(5):sbaf174. doi: 10.1093/schbul/sbaf174 (PMC13645815; doi:10.1093/schbul/sbaf174)
Supplement: Supplementary file 1 [file psymetric_can-supmat-25052025_sbaf174.docx]

**Supplementary Data :**

**Supplementary Methods :**

**Supplementary Figures :**

**Supplementary Tables :**

*Missing Data*

The amount and structure of missing data in the sample was assessed, and considered for multiple imputation using chained equations. To examine the pattern of missing data in the sample, we plotted a ‘missingness map’, showing a matrix of paired variables to show relationships between observed data (blue bars) and missing data (white bars) between the following variables at both baseline and follow-up. See below. bmi_0=baseline body mass index, Met_AP=metabolically active antipsychotic, smokingstatus_0=smoking status, tryglicerides_0=triglycerides, hdlcholesterol_0=high-density lipoprotein cholesterol, sex=sex, age_admn_0=age.

From the above missingness map plot, there appears a pattern of missing data such that when biochemical tests were not performed, all biochemical variables were missing together, as would be expected. There were no other common patterns of missing data between variables. Therefore, we did not find strong evidence that the data were ‘missing not at random’. However, because some patterns of missing data were evident in our sample, we only considered variables for imputation where they were <50% missing and had suitable auxiliary variables available for use as indicators of missingness, in order to reduce the impact of ‘missing not at random’ bias.

See the proportion of missing data per variable in each dataset below. We imputed 10 datasets in both samples. Box-and-Whisker and Density plots were used to check similarities of observed and imputed data (see below). The MetS outcome was calculated from the constituent variables after the multiple imputation step.

| **Variable** | Proportion missing |
| --- | --- |
| Sex | 0.001788909 |
| Race and Ethnicity | |
| White | 0.000000000 |
| Black or African American | 0.046511628 |
| East Asian/Aboriginal | 0.046511628 |
| Hispanic or Latino | 0.046511628 |
| South Asian | 0.046511628 |
| Other | 0.046511628 |
| Age | 0.000000000 |
| Smoking Status | 0.044722719 |
| Antipsychotic Prescription | 0.050089445 |
| SBP – Baseline | 0.066189624 |
| SBP – Follow-up | 0.033989267 |
| BMI – Baseline | 0.135957066 |
| BMI – Follow-up | 0.123434705 |
| Triglycerides – Baseline | 0.035778175 |
| Triglycerides – Follow-up | 0.012522361 |
| HDL – Baseline | 0.032200358 |
| HDL – Follow-up | 0.014311270 |

**Box-and-whisker plots to check similarity of observed (blue) and imputed (pink) data**

The box and whisker plots display the distribution of observed (blue) and imputed (pink) data for each imputed variable, across 10 imputed datasets.

**Strip plots to check similarity of observed (blue) and imputed (pink) data**

These one-dimensional scatterplots show the observed (blue) and imputed (pink) data points, for each imputed variable across 10 imputed datasets.

*Clinical Usefulness and Potential Cutoffs*

Decision curve analysis was used to assess clinical usefulness of PsyMetRiC by estimating net benefit across a range of feasible thresholds (i.e., the risk score at which an intervention would be deemed necessary). We will use a risk threshold upper-bound of 0.30, which represents around a one-in-three chance of developing MetS should nothing change, because it is unlikely that risk thresholds greater than that would be tolerated without intervention. Net benefit incorporates the consequences of the decisions made on the basis of an algorithm and is therefore preferable to related measures such as sensitivity and specificity.

Clinical Usefulness and Potential Cutoffs

Net benefit is a metric of true positives minus false positives, and is calculated as: sensitivity × prevalence – (1 – specificity) × (1 – prevalence) × w, where w is the outcome odds at a given risk threshold11. The risk threshold is the amount of tolerable risk before an intervention is deemed necessary. Net benefit incorporates the consequences of the decisions made on the basis of an algorithm, and is therefore preferable to related measures such as sensitivity and specificity alone. We also reported the standardized net benefit (net benefit / outcome prevalence) and related metrics (sensitivity and specificity) across a range of reasonable risk thresholds. We drew a decision curve plot which visualised and compared the clinical usefulness of the original PsyMetRiC algorithm (primary external validation analysis) vs the updated, recalibrated PsyMetRiC version, compared with intervening in all, or intervening in none. Classical decision theory proposes that at a chosen risk-threshold, the choice with the greatest net-benefit should be preferred.

**Supplementary Figures :**

**Supplementary Figure 1:** Pooled Predicted Probabilities for PsyMetRiC in the Analytic Sample Before and After Model Updating and Recalibration

B

A

D

C

A = PsyMetRiC full model in primary external validation analysis; B = PsyMetRiC full model after model updating and recalibration;

C = PsyMetRiC partial model in primary external validation analysis; D = PsyMetRiC partial model after model updating and recalibration

**Supplementary Figure 2:** Calibration Plots Across Imputed Datasets for PsyMetRiC in the Analytic Sample

**A-Full Model**

Calibration Plots of Primary External Validation Analysis

Calibration Plots After Model Updating and Recalibration

**B-Partial Model**

Calibration Plots of Primary External Validation Analysis

Calibration Plots After Model Updating and Recalibration

**Supplementary Figure 3:** Clinical Usefulness Across Imputed Datasets of PsyMetRiC in the Analytic Sample Before and After Model Updating and Recalibration

**A=PsyMetRiC Full-Model**

**B=PsyMetRiC Partial-Model**

**Supplementary Table 1:** Pooled Net Benefit and Standardized Net Benefit of The PsyMetRiC Full-Model (Original and Updated/Recalibrated Versions) in the Analytic Sample Across a Range of Feasible Risk Thresholds

| **Risk Threshold** | **Net Benefit Full (Updated/Recalibrated) + 95% CI** | **Net Benefit Full (Original) + 95% CI** | **Standardized Net Benefit (Updated/Recalibrated) + 95% CI** | **Standardized Net Benefit (Original) + 95% CI** |
| --- | --- | --- | --- | --- |
| **0.00** | 0.172 (0.134-0.204) | 0.172 (0.147-0.204) | 1 (1-1) | 1 (1-1) |
| **0.01** | 0.163 (0.126-0.196) | 0.163 (0.138-0.196) | 0.952 (0.936-0.961) | 0.951 (0.941-0.961) |
| **0.02** | 0.155 (0.117-0.188) | 0.155 (0.129-0.188) | 0.905 (0.874-0.923) | 0.902 (0.881-0.92) |
| **0.03** | 0.146 (0.107-0.179) | 0.146 (0.121-0.18) | 0.849 (0.8-0.88) | 0.852 (0.822-0.88) |
| **0.04** | 0.14 (0.1-0.174) | 0.138 (0.112-0.171) | 0.813 (0.751-0.85) | 0.804 (0.765-0.841) |
| **0.05** | 0.131 (0.093-0.164) | 0.129 (0.101-0.163) | 0.76 (0.691-0.807) | 0.752 (0.69-0.798) |
| **0.06** | 0.126 (0.088-0.159) | 0.12 (0.093-0.152) | 0.734 (0.656-0.784) | 0.699 (0.631-0.755) |
| **0.07** | 0.118 (0.08-0.151) | 0.115 (0.088-0.147) | 0.687 (0.592-0.753) | 0.667 (0.58-0.736) |
| **0.08** | 0.109 (0.072-0.141) | 0.111 (0.084-0.145) | 0.634 (0.526-0.711) | 0.648 (0.558-0.725) |
| **0.09** | 0.104 (0.068-0.136) | 0.103 (0.076-0.139) | 0.607 (0.489-0.683) | 0.598 (0.5-0.685) |
| **0.10** | 0.096 (0.06-0.126) | 0.1 (0.073-0.136) | 0.557 (0.429-0.646) | 0.581 (0.485-0.669) |
| **0.11** | 0.089 (0.054-0.116) | 0.09 (0.064-0.123) | 0.516 (0.391-0.616) | 0.523 (0.423-0.613) |
| **0.12** | 0.087 (0.053-0.116) | 0.088 (0.062-0.122) | 0.507 (0.375-0.604) | 0.514 (0.405-0.611) |
| **0.13** | 0.083 (0.05-0.111) | 0.087 (0.06-0.12) | 0.483 (0.355-0.576) | 0.506 (0.387-0.598) |
| **0.14** | 0.082 (0.049-0.11) | 0.08 (0.053-0.108) | 0.477 (0.343-0.575) | 0.466 (0.354-0.561) |
| **0.15** | 0.077 (0.046-0.107) | 0.078 (0.053-0.106) | 0.45 (0.317-0.55) | 0.456 (0.334-0.55) |
| **0.16** | 0.07 (0.039-0.097) | 0.078 (0.052-0.107) | 0.405 (0.266-0.504) | 0.452 (0.33-0.545) |
| **0.17** | 0.071 (0.04-0.097) | 0.077 (0.051-0.105) | 0.412 (0.271-0.511) | 0.451 (0.321-0.543) |
| **0.18** | 0.068 (0.038-0.096) | 0.064 (0.039-0.091) | 0.398 (0.265-0.502) | 0.374 (0.253-0.481) |
| **0.19** | 0.066 (0.036-0.094) | 0.064 (0.04-0.093) | 0.385 (0.254-0.492) | 0.373 (0.246-0.492) |
| **0.20** | 0.068 (0.038-0.095) | 0.06 (0.037-0.087) | 0.393 (0.264-0.5) | 0.352 (0.225-0.46) |
| **0.21** | 0.061 (0.031-0.088) | 0.052 (0.031-0.078) | 0.352 (0.217-0.441) | 0.304 (0.19-0.408) |
| **0.22** | 0.053 (0.024-0.082) | 0.042 (0.021-0.066) | 0.309 (0.157-0.41) | 0.243 (0.13-0.342) |
| **0.23** | 0.049 (0.018-0.079) | 0.039 (0.019-0.061) | 0.285 (0.121-0.388) | 0.23 (0.12-0.323) |
| **0.24** | 0.05 (0.019-0.079) | 0.039 (0.019-0.06) | 0.291 (0.13-0.398) | 0.226 (0.111-0.322) |
| **0.25** | 0.049 (0.02-0.075) | 0.035 (0.016-0.055) | 0.285 (0.131-0.387) | 0.205 (0.107-0.301) |
| **0.26** | 0.042 (0.012-0.069) | 0.033 (0.014-0.052) | 0.243 (0.082-0.357) | 0.192 (0.09-0.281) |
| **0.27** | 0.036 (0.006-0.063) | 0.032 (0.013-0.051) | 0.211 (0.043-0.321) | 0.184 (0.09-0.277) |
| **0.28** | 0.034 (0.005-0.059) | 0.027 (0.009-0.043) | 0.201 (0.032-0.306) | 0.155 (0.066-0.242) |
| **0.29** | 0.031 (0.007-0.053) | 0.02 (0.006-0.037) | 0.178 (0.052-0.287) | 0.115 (0.043-0.202) |
| **0.30** | 0.028 (0.003-0.051) | 0.017 (0.004-0.034) | 0.164 (0.026-0.272) | 0.098 (0.026-0.184) |

^a^Different risk thresholds can be selected depending on the proposed intervention (i.e. balancing the risk/benefit of exposing false positives to an intervention to benefit the most true positives) and patient or clinician preference.

^b^Standardised net benefit is calculated as net benefit/outcome prevalence, showing the proportion of improvement in net benefit at the selected risk threshold.

**Supplementary Table 2:** Pooled Net Benefit and Standardized Net Benefit of The PsyMetRiC Partial-Model (Original and Updated/Recalibrated Versions) in the Analytic Sample Across a Range of Feasible Risk Thresholds

| **Risk Threshold** | **Net Benefit Partial (Updated/Recalibrated) + 95% CI** | **Net Benefit Partial (Original) + 95% CI** | **Standardized Net Benefit (Updated/Recalibrated) + 95% CI** | **Standardized Net Benefit (Original) + 95% CI** |
| --- | --- | --- | --- | --- |
| **0.00** | 0.172 (0.143-0.199) | 0.172 (0.141-0.204) | 1 (1-1) | 1 (1-1) |
| **0.01** | 0.163 (0.134-0.19) | 0.163 (0.133-0.196) | 0.951 (0.94-0.959) | 0.951 (0.939-0.961) |
| **0.02** | 0.155 (0.126-0.182) | 0.155 (0.124-0.188) | 0.903 (0.88-0.919) | 0.902 (0.876-0.92) |
| **0.03** | 0.147 (0.117-0.174) | 0.146 (0.115-0.179) | 0.854 (0.819-0.877) | 0.851 (0.812-0.879) |
| **0.04** | 0.139 (0.11-0.167) | 0.137 (0.106-0.171) | 0.809 (0.766-0.841) | 0.799 (0.747-0.837) |
| **0.05** | 0.129 (0.1-0.159) | 0.128 (0.096-0.162) | 0.753 (0.698-0.801) | 0.747 (0.681-0.795) |
| **0.06** | 0.123 (0.094-0.153) | 0.119 (0.087-0.153) | 0.716 (0.648-0.77) | 0.693 (0.613-0.751) |
| **0.07** | 0.11 (0.082-0.138) | 0.11 (0.077-0.144) | 0.643 (0.557-0.71) | 0.639 (0.546-0.708) |
| **0.08** | 0.105 (0.075-0.132) | 0.101 (0.068-0.135) | 0.61 (0.511-0.68) | 0.589 (0.482-0.664) |
| **0.09** | 0.096 (0.065-0.121) | 0.09 (0.057-0.122) | 0.56 (0.448-0.639) | 0.526 (0.399-0.612) |
| **0.10** | 0.092 (0.062-0.117) | 0.081 (0.048-0.114) | 0.536 (0.419-0.626) | 0.473 (0.332-0.57) |
| **0.11** | 0.085 (0.057-0.109) | 0.074 (0.038-0.107) | 0.496 (0.383-0.584) | 0.428 (0.266-0.53) |
| **0.12** | 0.081 (0.053-0.107) | 0.064 (0.03-0.1) | 0.473 (0.358-0.561) | 0.372 (0.203-0.482) |
| **0.13** | 0.07 (0.041-0.097) | 0.057 (0.023-0.091) | 0.41 (0.272-0.508) | 0.334 (0.154-0.451) |
| **0.14** | 0.062 (0.035-0.088) | 0.055 (0.021-0.087) | 0.364 (0.233-0.477) | 0.319 (0.143-0.438) |
| **0.15** | 0.062 (0.034-0.089) | 0.053 (0.017-0.086) | 0.359 (0.233-0.475) | 0.306 (0.123-0.431) |
| **0.16** | 0.06 (0.033-0.084) | 0.044 (0.006-0.078) | 0.347 (0.21-0.451) | 0.254 (0.04-0.389) |
| **0.17** | 0.053 (0.024-0.079) | 0.041 (0.005-0.075) | 0.307 (0.165-0.424) | 0.239 (0.033-0.376) |
| **0.18** | 0.052 (0.025-0.074) | 0.037 (0.003-0.072) | 0.303 (0.159-0.404) | 0.217 (0.024-0.352) |
| **0.19** | 0.053 (0.026-0.075) | 0.037 (0.003-0.073) | 0.31 (0.17-0.41) | 0.214 (0.019-0.346) |
| **0.20** | 0.049 (0.023-0.074) | 0.038 (0.004-0.072) | 0.286 (0.153-0.395) | 0.221 (0.03-0.353) |
| **0.21** | 0.047 (0.021-0.069) | 0.024 (-0.008-0.059) | 0.274 (0.133-0.369) | 0.141 (-0.056-0.299) |
| **0.22** | 0.042 (0.018-0.065) | 0.019 (-0.012-0.052) | 0.247 (0.117-0.352) | 0.111 (-0.086-0.261) |
| **0.23** | 0.039 (0.015-0.062) | 0.021 (-0.009-0.055) | 0.226 (0.1-0.323) | 0.123 (-0.066-0.274) |
| **0.24** | 0.032 (0.006-0.055) | 0.017 (-0.011-0.049) | 0.184 (0.041-0.281) | 0.101 (-0.082-0.262) |
| **0.25** | 0.03 (0.004-0.052) | 0.017 (-0.014-0.047) | 0.177 (0.027-0.283) | 0.097 (-0.1-0.247) |
| **0.26** | 0.03 (0.004-0.052) | 0.016 (-0.013-0.046) | 0.172 (0.026-0.285) | 0.094 (-0.089-0.249) |
| **0.27** | 0.029 (0.005-0.053) | 0.016 (-0.015-0.044) | 0.169 (0.03-0.281) | 0.091 (-0.1-0.239) |
| **0.28** | 0.029 (0.004-0.051) | 0.02 (-0.009-0.048) | 0.169 (0.026-0.276) | 0.114 (-0.056-0.264) |
| **0.29** | 0.027 (0.001-0.051) | 0.018 (-0.01-0.047) | 0.158 (0.009-0.274) | 0.107 (-0.063-0.263) |
| **0.30** | 0.027 (0.003-0.05) | 0.018 (-0.01-0.049) | 0.159 (0.016-0.268) | 0.107 (-0.062-0.252) |

^a^Different risk thresholds can be selected depending on the proposed intervention (i.e. balancing the risk/benefit of exposing false positives to an intervention to benefit the most true positives) and patient or clinician preference.

^b^Standardised net benefit is calculated as net benefit/outcome prevalence, showing the proportion of improvement in net benefit at the selected risk threshold.

| **^More Metabolically-Active Antipsychotics^** | **^Less Metabolically-Active Antipsychotics^** |
| --- | --- |
| ^Olanzapine^ | ^Aripiprazole^ |
| ^Quetiapine^ | ^Haloperidol^ |
| ^Risperidone^ | ^Lurasidone^ |
| ^Paliperidone^ | ^Ziprasdone^ |
| ^Clozapine^ | ^Flupenthixol^ |
| ^Asenapine^ | ^Brexpiprazole^ |

**Supplementary Table 3: Classification of More Metabolically-Active Antipsychotics**

^This table comprises all antipsychotics prescribed for participants/patients in the analytic sample^

**Supplementary Table 4 : Coefficients for Updated and Recalibrated PsyMetRiC^a^**

|  | **Full-Model** | **Partial-Model** |
| --- | --- | --- |
| **Intercept** | -6.84247617 | -6.553710864 |
| **Slope** | 1.299551543 | 1.012065547 |
| **Race and Ethnicity** | | |
| **Black or African American** | 0.322993879 | 0.105445104 |
| **East Asian/Aboriginal** | -0.891008005 | -0.919120171 |
| **Hispanic or Latino** | -0.157486705 | -0.314191869 |
| **South Asian** | 0.244196907 | 0.017563594 |
| **Other** | 0.697311353 | 0.779604311 |

^a^Other coefficients remain the same as the original PsyMetRiC algorithm

**Supplementary Table 5 : Original PsyMetRiC algorithm (UK) coefficients after shrinkage for optimism**

| **PsyMetRiC Predictor** | **Full-Model** | **Partial-Model** |
| --- | --- | --- |
| Intercept | -6.439813 | -6.973829 |
| Age in years (continuous) | 0.006233226 | 0.00633115 |
| Black/African-Caribbean Ethnicity (yes/no) | 0.004258861 | 0.07548129 |
| Asian / Other Ethnicity (yes/no) | 0.211217746 | 0.29285950 |
| Male Sex (yes/no) | 0.222300765 | 0.31460036 |
| Body Mass Index (BMI) (kg/m^2^) (continuous) | 0.141186241 | 0.16912161 |
| Current Smoking Status (smoker, non-smoker) | 0.153691193 | 0.24751854 |
| Prescribed a Metabolically-Active Antipsychotic (yes/no) | 0.497552758 | 0.60013558 |
| High-Density Lipoprotein (HDL) (mmol/L) (continuous) | -0.399013329 | ^-^ |
| Triglycerides (mmol/L) (continuous) | 0.343528440 | ^-^ |

**Supplementary Table 6 : TRIPOD AI - Checklist**

| **Section/Topic Item Development Checklist item**  **/ evaluation**^1^ | | | | | | | **Reported on page** |
| --- | --- | --- | --- | --- | --- | --- | --- |
| **TITLE** | | | | | | |  |
| *Title* | | 1 | | D;E | Identify the study as developing or evaluating the performance of a multivariable prediction model, the target population, and the outcome to be predicted | | 3 |
| **ABSTRACT** | | | | | | | |
| *Abstract* | | 2 | | D;E | See TRIPOD+AI for Abstracts checklist | | 3 |
| **INTRODUCTION** | | | | | | | |
| *Background* | | 3a | | D;E | Explain the healthcare context (including whether diagnostic or prognostic) and rationale for developing or evaluating the prediction model, including references to existing models | | 5 |
|  |  | 3b | | D;E | Describe the target population and the intended purpose of the prediction model in the context of the care pathway, including its intended users (e.g., healthcare professionals, patients, public) | | 5 |
|  |  | 3c | | D;E | Describe any known health inequalities between sociodemographic groups | | 5 |
| *Objectives* | | 4 | | D;E | Specify the study objectives, including whether the study describes the development or validation of a prediction model (or both) | | 6 |
| **METHODS** | | | | | | | |
| *Data* | | 5a | | D;E | Describe the sources of data separately for the development and evaluation datasets (e.g., randomised trial, cohort, routine care or registry data), the rationale for using these data, and representativeness of the data | | 6, 11 |
|  |  | 5b | | D;E | Specify the dates of the collected participant data, including start and end of participant accrual; and, if applicable, end of follow-up | | 6 |
| *Participants* | | 6a | | D;E | Specify key elements of the study setting (e.g., primary care, secondary care, general population)  including the number and location of centres | | 6 |
|  |  | 6b | | D;E | Describe the eligibility criteria for study participants | | 6 |
|  |  | 6c | | D;E | Give details of any treatments received, and how they were handled during model development or evaluation, if relevant | | 6 |
| *Data preparation* | | 7 | | D;E | Describe any data pre-processing and quality checking, including whether this was similar across  relevant sociodemographic groups | | 7 |
| *Outcome* | | 8a | | D;E | Clearly define the outcome that is being predicted and the time horizon, including how and when assessed, the rationale for choosing this outcome, and whether the method of outcome assessment is  consistent across sociodemographic groups | | 6 |
|  |  | 8b | | D;E | If outcome assessment requires subjective interpretation, describe the qualifications and demographic characteristics of the outcome assessors | | NA |
|  |  | 8c | | D;E | Report any actions to blind assessment of the outcome to be predicted | | NA |
| *Predictors* | | 9a | | D | Describe the choice of initial predictors (e.g., literature, previous models, all available predictors) and  any pre-selection of predictors before model building | | NA |
|  |  | 9b | | D;E | Clearly define all predictors, including how and when they were measured (and any actions to blind assessment of predictors for the outcome and other predictors) | | 6 |
|  |  | 9c | | D;E | If predictor measurement requires subjective interpretation, describe the qualifications and demographic characteristics of the predictor assessors | | NA |
| *Sample size* | | 10 | | D;E | Explain how the study size was arrived at (separately for development and evaluation), and justify that  the study size was sufficient to answer the research question. Include details of any sample size calculation | | 7 |
| *Missing data* | | 11 | | D;E | Describe how missing data were handled. Provide reasons for omitting any data | | 7 |
| *Analytical methods* | | 12a | | D | Describe how the data were used (e.g., for development and evaluation of model performance) in the analysis, including whether the data were partitioned, considering any sample size requirements | | NA |
|  |  | 12b | | D | Depending on the type of model, describe how predictors were handled in the analyses (functional form,  rescaling, transformation, or any standardisation). | | NA |
|  |  | 12c | | D | Specify the type of model, rationale^2^, all model-building steps, including any hyperparameter tuning,  and method for internal validation | | NA |
|  |  | 12d | | D;E | Describe if and how any heterogeneity in estimates of model parameter values and model performance was handled and quantified across clusters (e.g., hospitals, countries). See TRIPOD-Cluster for  additional considerations^3^ | | 7 |
|  |  | 12e | | D;E | Specify all measures and plots used (and their rationale) to evaluate model performance (e.g., discrimination, calibration, clinical utility) and, if relevant, to compare multiple models | | 7 |
|  |  | 12f | | E | Describe any model updating (e.g., recalibration) arising from the model evaluation, either overall or for particular sociodemographic groups or settings | | 7 |
|  |  | 12g | | E | For model evaluation, describe how the model predictions were calculated (e.g., formula, code, object, application programming interface) | | 7 |
| *Class imbalance* | | 13 | | D;E | If class imbalance methods were used, state why and how this was done, and any subsequent methods to  recalibrate the model or the model predictions | | NA |
| *Fairness* | | 14 | | D;E | Describe any approaches that were used to address model fairness and their rationale | | 7 |
| *Model output* | | 15 | | D | Specify the output of the prediction model (e.g., probabilities, classification). Provide details and  rationale for any classification and how the thresholds were identified | | NA |
| *Training versus*  *evaluation* | | 16 | | D;E | Identify any differences between the development and evaluation data in healthcare setting, eligibility  criteria, outcome, and predictors | | Table 1 |
| *Ethical approval* | | 17 | | D;E | Name the institutional research board or ethics committee that approved the study and describe the participant-informed consent or the ethics committee waiver of informed consent | | 8 |
| **OPEN SCIENCE** | | | | | | | |
| *Funding* | 18a | | D;E | | | Give the source of funding and the role of the funders for the present study | 8 |
| *Conflicts of interest* | 18b | | D;E | | | Declare any conflicts of interest and financial disclosures for all authors | 3 |
| *Protocol* | 18c | | D;E | | | Indicate where the study protocol can be accessed or state that a protocol was not prepared | 6 |
| *Registration* | 18d | | D;E | | | Provide registration information for the study, including register name and registration number, or state  that the study was not registered | 6 |
| *Data sharing* | 18e | | D;E | | | Provide details of the availability of the study data | 12 |
| *Code sharing* | 18f | | D;E | | | Provide details of the availability of the analytical code^4^ | 12 |
| **PATIENT & PUBLIC INVOLVEMENT** | | | | | | | |
| *Patient & Public Involvement* | 19 | | D;E | | | Provide details of any patient and public involvement during the design, conduct, reporting, interpretation, or dissemination of the study or state no involvement. | 6 |
| **RESULTS** | | | | | | | |
| *Participants* | 20a | | D;E | | | Describe the flow of participants through the study, including the number of participants with and without the outcome and, if applicable, a summary of the follow-up time. A diagram may be helpful. | 8 |
|  | 20b | | D;E | | | Report the characteristics overall and, where applicable, for each data source or setting, including the key dates, key predictors (including demographics), treatments received, sample size, number of outcome events, follow-up time, and amount of missing data. A table may be helpful. Report any  differences across key demographic groups. | 8 |
|  | 20c | | E | | | For model evaluation, show a comparison with the development data of the distribution of important predictors (demographics, predictors, and outcome). | 8 |
| *Model development* | 21 | | D;E | | | Specify the number of participants and outcome events in each analysis (e.g., for model development, hyperparameter tuning, model evaluation) | 8 |
| *Model specification* | 22 | | D | | | Provide details of the full prediction model (e.g., formula, code, object, application programming interface) to allow predictions in new individuals and to enable third-party evaluation and implementation, including any restrictions to access or re-use (e.g., freely available, proprietary)^5^ | 7 |
| *Model performance* | 23a | | D;E | | | Report model performance estimates with confidence intervals, including for any key subgroups (e.g., sociodemographic). Consider plots to aid presentation. | 8 |
|  | 23b | | D;E | | | If examined, report results of any heterogeneity in model performance across clusters. See TRIPOD  Cluster for additional details^3^. | NA |
| *Model updating* | 24 | | E | | | Report the results from any model updating, including the updated model and subsequent performance | 9 |
| **DISCUSSION** | | | | | | | |
| *Interpretation* | 25 | | D;E | | | Give an overall interpretation of the main results, including issues of fairness in the context of the  objectives and previous studies | 9, 10 |
| *Limitations* | 26 | | D;E | | | Discuss any limitations of the study (such as a non-representative sample, sample size, overfitting, missing data) and their effects on any biases, statistical uncertainty, and generalizability | 10, 11 |
| *Usability of the model in the context of current care* | 27a | | D | | | Describe how poor quality or unavailable input data (e.g., predictor values) should be assessed and handled when implementing the prediction model | NA |
|  | 27b | | D | | | Specify whether users will be required to interact in the handling of the input data or use of the model,  and what level of expertise is required of users | NA |
|  | 27c | | D;E | | | Discuss any next steps for future research, with a specific view to applicability and generalizability of  the model | 12 |
